# Inferences about the population history of *Rangifer tarandus* from Y chromosome and mtDNA phylogenies

**DOI:** 10.1002/ece3.11573

**Published:** 2024-06-10

**Authors:** Elif Bozlak, Kisun Pokharel, Melak Weldenegodguad, Antti Paasivaara, Florian Stammler, Knut H. Røed, Juha Kantanen, Barbara Wallner

**Affiliations:** ^1^ Department of Biomedical Sciences, Institute of Animal Breeding and Genetics University of Veterinary Medicine Vienna Vienna Austria; ^2^ Vienna Graduate School of Population Genetics University of Veterinary Medicine Vienna Vienna Austria; ^3^ Natural Resources Institute Finland Jokioinen Finland; ^4^ Natural Resources Institute Finland Oulu Finland; ^5^ Arctic Centre University of Lapland Rovaniemi Finland; ^6^ Department of Preclinical Sciences and Pathology Norwegian University of Life Sciences Ås Norway

**Keywords:** caribou, haplotypes, population genetics, reindeer, uniparental markers

## Abstract

Reindeer, called caribou in North America, has a circumpolar distribution and all extant populations belong to the same species (*Rangifer tarandus*). It has survived the Holocene thanks to its immense adaptability and successful coexistence with humans in different forms of hunting and herding cultures. Here, we examine the paternal and maternal history of *Rangifer* based on robust Y‐chromosomal and mitochondrial DNA (mtDNA) trees representing Eurasian tundra reindeer, Finnish forest reindeer, Svalbard reindeer, Alaska tundra caribou, and woodland caribou. We first assembled Y‐chromosomal contigs, representing 1.3 Mb of single‐copy Y regions. Based on 545 Y‐chromosomal and 458 mtDNA SNPs defined in 55 males, maximum parsimony trees were created. We observed two well separated clades in both phylogenies: the “EuroBeringian clade” formed by animals from Arctic Islands, Eurasia, and a few from North America and the “North American clade” formed only by caribou from North America. The time calibrated Y tree revealed an expansion and dispersal of lineages across continents after the Last Glacial Maximum. We show for the first time unique paternal lineages in Svalbard reindeer and Finnish forest reindeer and reveal a circumscribed Y haplogroup in Fennoscandian tundra reindeer. The Y chromosome in domesticated reindeer is markedly diverse indicating that several male lineages have undergone domestication and less intensive selection on males. This study places *R. tarandus* onto the list of species with resolved Y and mtDNA phylogenies and builds the basis for studies of the distribution and origin of paternal and maternal lineages in the future.

## Graphical abstract

We established for the first time comprehensive analysis of Y and mtDNA lineages in Rangifer tarandus, representing reindeer and caribou across North America, Eurasia, and the Arctic Islands. Our results highlight migrations over long distances after the Last Glacial Maximum, subsequent recolonizations, and human‐mediated trajectories, especially the domestication process.

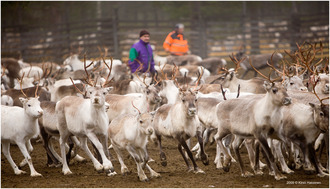

## 1 INTRODUCTION

The male‐specific region of mammalian Y chromosomes, commonly known as MSY, is inherited without recombination from the father to his sons. This strictly paternally inherited part of the genome holds crucial information about the male‐mediated population history, complementary to the maternally inherited mitochondrial DNA (mtDNA). Hence, MSY haplotype (HT) trees allow inferences about a species' evolutionary past, like the spread of lineages, historical migrations, admixture between populations and colonization events (Jobling & Tyler‐Smith, 2017; Poznik et al., 2016).

The MSY is most well studied in hominid lineages and findings there underline its valuable information content (Barbieri et al., 2016; Hallast et al., 2016; Lippold et al., 2014) which also advances MSY analysis in mammals. But the complex sequence structure of the chromosome still hampers the investigation of MSY sequence variation. The MSY consists mainly of repetitive sequences, its genic regions are largely ampliconic, and the remaining parts are homologous to the X chromosome to varying degrees (Rhie et al., 2023; Skaletsky et al., 2003). However, recent achievements, like refined Y assembly techniques (Felkel, Vogl, et al., 2019; Hallast et al., 2023; Rhie et al., 2023), enhanced data analysis methods (Jobling & Tyler‐Smith, 2017; Martiniano et al., 2022) and extensive sequencing datasets (Bozlak et al., 2023; Deng et al., 2020; Hallast et al., 2023) made the mammalian MSY a widely used marker to address patrilineal population dynamics.

Overall, MSY studies in mammals are conducted over a range of evolutionary time scales, from comparative analysis among closely related taxa (as shown in bears Bidon et al., 2014; and apes Hallast et al., 2016), to investigations of recent population dynamics, especially in domesticated stock. In domestics, the most pronounced MSY signals are male bottlenecks during the domestication process, the distribution of domestic lineages, admixture between wild and domestic populations, and the massive decline of MSY diversity due to amplification of few selected males in recent intensive breeding regimes. This has been shown in horses (Remer et al., 2022; Wallner et al., 2017), sheep (Deng et al., 2020), goats (Nijman et al., 2022; Xiao et al., 2021), pigs (Ai et al., 2021; Guirao‐Rico et al., 2018), cattle (Chen, Cai, et al., 2018; Chen, Zhou, et al., 2018; Escouflaire & Capitan, 2021; Ganguly et al., 2020), and dogs (Oetjens et al., 2018; Smeds et al., 2019).

*Rangifer tarandus*, commonly known as caribou in North America and reindeer in Eurasia, holds a unique position among mammals due to its circumpolar distribution (Dussex et al., 2023; Kvie et al., 2016), its migratory behavior, the adaptation to cold environment (Lin et al., 2019), and having domesticated and wild animals in close contact (Anderson et al., 2017; Harding, 2022). The species likely originated in Beringia, approximately 1.6 million years ago (Harington, 1999) and with changing climatic conditions during and after the ice age, numerous *Rangifer* lineages emerged (Polfus et al., 2017) out of which several got extinct (Banfield, 1961; Harding, 2022). Today, *Rangifer* is spread across Northern Eurasia, North America and Arctic Islands, and those are classified into several subspecies and ecotypes. Genetic studies based on autosomal and mtDNA revealed significant differentiation between caribou of North America (more specifically Canada) and the so‐called “EuroBeringian” *Rangifer* populations of Eurasia, Arctic Islands and Alaska (Dussex et al., 2023; Flagstad & Røed, 2003; Harding, 2022; Yannic et al., 2014). This divergence was attributed to the North American ice sheet, which served as a barrier between the caribou in the South and the EuroBeringian lineages in the North from 21 to 10 thousand years ago (kya) (Yannic et al., 2014).

Among EuroBeringian *Rangifer* populations genetic data indicated a clear demarcation of the Svalbard reindeer (*R. t. platyrhynchus*), and a distinction of the two Eurasian *Rangifer* subspecies – the Eurasian tundra reindeer (*R. t. tarandus*) and the Finnish forest reindeer (*R. t. fennicus*; Dussex et al., 2023; Heino et al., 2021; Kvie et al., 2016; Pokharel et al., 2023; Weldenegodguad et al., 2020, 2023). Within Eurasian tundra reindeer, mtDNA and autosomal data revealed a clear separation of the Fennoscandian populations, and this was interpreted as the unique ancestry of Fennoscandian reindeer from an isolated refugium (Flagstad & Røed, 2003; Pokharel et al., 2023; Weldenegodguad et al., 2020).

Furthermore, the history of reindeer is closely intertwined with that of humans in the Eurasian Arctic region. *Rangifer* was probably the key species for human survival and colonization of the area, with initially hunted and later domesticated reindeer providing meat, clothing, housing, and transportation. The first reindeer herders primarily lived in a hunter‐gatherer economy and domesticated reindeer were mainly used for transportation and as decoy animals to attract wild reindeer (Losey et al., 2021). During the 18^th^ and 19^th^ centuries, there was a shift toward large‐scale extensive herding with herders subsisting primarily on domesticated animals. This pastoral transition led to new settlements and land use patterns across large portions of Northern Eurasia, and it is considered to be one of the most fundamental social transformations that ever took place in the Eurasian Arctic region (Krupnik, 1993; Røed et al., 2020).

*Rangifer* has been an indispensable source of life in the Arctic and continues to be so for the northernmost societies on our planet. However, industrialization, increased human settlement in the last centuries, as well as global warming have let the habitat for reindeer decrease. Consequently, also the conditions for continuing reindeer herding and hunting as a livelihood become ever more challenging. In order to best preserve the current *Rangifer* populations, it is important to know their current genetic makeup, but it is also crucial to understand the origin and historical development of the species.

Due to the lack of recombination, Y and mtDNA phylogenies retain information about the sequential accumulation of genetic variation. They therefore offer the opportunity to clearly separate ancient colonization events from subsequent overlying migrations, in particular recent introgressions. Uniparental markers are particularly informative in a migratory species such as *Rangifer*, where diverse patterns of fragmentation and colonization contributed to extant populations. However, findings from these two markers, the Y and the mtDNA, are often in discrepancy (Achilli et al., 2012; Bidon et al., 2014; Deng et al., 2020; Felkel et al., 2019), which can result from different mating dynamics of the two sexes, different dispersal rates or sex specific selection, but also drift and differences in mutations rates (Di Lorenzo et al., 2016; Frantz et al., 2020; Underhill & Kivisild, 2007). The commonly observed different results in Y and mtDNA highlight that a proper investigation of both uniparental markers is needed for a comprehensive view on the phylogeographic history of populations. But while the mtDNA landscape of *Rangifer tarandus* is already comprehensively described (Flagstad & Røed, 2003; Hold et al., 2024; Taylor et al., 2021; Yannic et al., 2014), the MSY variation is not yet studied.

Here, we established robust analysis of Y lineages in reindeer by analyzing sequencing data from 55 male reindeer and caribou collected across North America, Eurasia, and the Arctic Islands, representing five subspecies. Our findings provide first insights into the male demography of reindeer and contribute to a deeper understanding of the population history of these animals. We contrast Y and mtDNA lineages in our dataset and discuss our findings in the context of early migrations following the Last Glacial Maximum (LGM), subsequent recolonization patterns, and human‐mediated trajectories, especially the domestication process.

## 2 MATERIALS AND METHODS

### 2.1 Whole genome sequencing data

Publicly available whole genome sequencing (WGS) data from 55 male and five female *Rangifer tarandus* (reindeer and caribou) specimens and a male moose (*Alces alces americanus*) were downloaded from repositories. The collection included 33 male and four female Eurasian tundra reindeer (*R. t. tarandus*), one male and one female Svalbard reindeer (*R. t. platyrhunchus*), two male Alaska tundra caribou (*R. t. granti*), four male Finnish forest reindeer (*R. t. fennicus*), and 15 male woodland caribou (*R. t. caribou*). Out of the 33 Eurasian tundra reindeer, 27 originated from domestic herds in Fennoscandia, Western and Eastern Russia (Arkhangelsk and Yakutia). Details on samples and raw data used for analysis are given in Table S1.

No information on the individual's sex was available for the caribou WGS data published in PRJNA846266. The fastq reads for the 142 specimens in that Bioproject were blasted against the *R. tarandus SRY* gene mRNA sequence (AB247629.1) with blastn (Altschul et al., 1990) default settings. The sex of each individual was determined by counting the number of reads with at least 95% similarity to the *SRY* coding region. Nine samples, with >13 reads matching and having at least 4× sequencing coverage, estimated based on the number of total bases divided by the size of reindeer genome assembly (2.9 Gb), were selected (Table S1).

Quality check of paired‐end WGS data was performed using fastqc (v. 0.11.5; Andrews, 2010). The default parameters of fastp (v. 0.21.0; Chen, Cai, et al., 2018; Chen, Zhou, et al., 2018) were utilized to trim the reads for all 61 samples.

### 2.2 Assembling Y‐chromosomal contigs

Three male Eurasian tundra reindeer (Fi‐D‐ETR‐6, Fi‐D‐ETR‐8, Fi‐D‐ETR‐10) were selected for assembling Y‐chromosomal contigs. First, sequencing reads from these individuals were merged, resulting in a total of 431,256,693 × 2 reads. Those reads were mapped to the female reindeer assembly (Li et al., 2017) with bwa mem (v. bwa‐0.7.17; Li, 2013) with default parameters. Unmapped reads were collected with samtools (v. samtools‐1.10 parameters ‐f 12 ‐F 256; Li et al., 2009) and two fastq files were generated from the resulting bam using bedtools (Quinlan & Hall, 2010) bamtofastq (v. bedtools2). SPAdes (v. SPAdes‐3.14.0; Bankevich et al., 2012) was employed to assemble the 3,349,523 × 2 unmapped reads in careful mode. Only contigs longer than 300 base pairs (bp) were considered for further analysis.

To distinguish the Y‐chromosomal contigs from autosomal contaminants, we mapped WGS data from 11 male and five female reindeer (samples indicated in Table S1) to the assembled 53,855 contigs using bwa aln (Li & Durbin, 2009) with “‐n 0.02 ‐l 200” parameters. We just retained reads mapped properly paired, and filtered out duplicate reads, and reads having less than 20 mapping quality using samtools. Coverage statistics were calculated from the filtered bam files for all samples with bedtools genomecov (v. bedtools2). For each contig, we calculated the coverage percentage for every individual. Then the mean coverage percentage for each contig was determined for males and females. To distinguish the true Y candidates among all contigs, a linear regression model was run between mean coverage percentage values in males versus females for each contig in R (v. 3.6.2; R Core Team, 2021). The function “augment” from the “broom” package (Robinson, 2014) was used to obtain the residual values for each observation and contigs were categorized as Y candidate (if .resid < −35 and .std.resid < 0) or not Y candidate.

### 2.3 Classifying Y contigs into scY, mcY, and nonY regions

The filtered bam files from 11 males and 5 females described above were used as input information for the classification of single‐copy Y (scY), multi‐copy Y (mcY), and not MSY (nonY) windows on the Y candidate contigs. The classification based on mapping depth in males and females was conducted with a probabilistic model published by Felkel, Vogl, et al. (2019). As sequencing depth differs among individuals (Table S1), each window's mean coverage was first normalized for the individuals' diploid coverage. To obtain autosomal coverages for normalization, coverage depth values on potential autosomal contigs in the initial assembly were used. Contigs were considered autosomal if they (i) were covered at least 75% in both males and females and (ii) exhibited a difference of no more than 0.01 male‐to‐female mean coverage ratio. In total, we selected 5478 autosomal (total length 4,990,511 bp) and 1324 Y‐chromosomal candidate contigs for the model.

For classification, the selected contigs (Y and autosomal) were split into 50 bp windows and the per‐site mean coverage of each window in the 11 male and 5 female reindeer was determined using bedtools. For each reindeer, the mode of the distribution of the mean coverages on the autosomal contigs was calculated (Table S1). This value was used to normalize the mean coverage per window in the Y contigs, such that a relative coverage of one corresponds to a diploid state. To implement female background coverage on Y contigs the mode of the mean coverage values on the Y contigs for female samples was included in the model (details and R‐codes are given in Felkel, Vogl, et al., 2019).

The probability of being MSY or nonY was calculated for each window by the model, with windows having a probability value greater than 0.5 were classified as MSY. Among the MSY windows, the classification of scY and mcY regions was based on the normalized mapping coverage in males. In this study, 1320 contigs, which mainly carried MSY windows (example Figure S1) form the “reindeerY1320” assembly (Table S2).

### 2.4 Placing reindeerY1320 contigs on reindeer Y chromosome (OX460344.1)

For a crosschecking male‐specificity and quality of the draft assembly, the reindeerY1320 contigs were aligned to a recently released full Y chromosome assembly from a Svalbard reindeer (GCA_949782905.1–OX460344.1; Dussex et al., 2023) with BLAST (v. 2.15.0+) and MUMmer (v. 3.23). blastn was used to align reindeerY1320 contigs and OX460344.1 with ‐perc_identity 95 parameter. Only alignments >100 bp were considered further (Table S3). In MUMmer, nucmer alignment was used in ‐maxmatch mode. nucmer alignment results were visualized through dot plot viewer (Dot, available at https://github.com/dnanexus‐archive/dot, last access April 21, 2024).

### 2.5 Y chromosome analysis

#### 2.5.1 Mapping and filtering of WGS data

Trimmed WGS reads of 40 male reindeer, 15 male caribou, and one male moose were mapped to the reindeerY1320 contigs with bwa aln using the parameters described above. Only mapped and properly paired reads were kept, and duplicate reads and reads having mapping quality less than 20 were removed with samtools. In addition, three male and three female reindeer (samples marked in Table S1) were mapped and filtered as described above using OX460344.1 as reference. Coverage estimations of the final bam files were conducted with samtools depth.

#### 2.5.2 Variant ascertainment

Variant calling was performed with freebayes (v. 1.3.2‐46‐g2c1e395; Garrison & Marth, 2012) in haploid mode in three separate attempts. First, variants were called among nine very low‐ (vLowDP = scY cov 1.7–2.6) and 40 low‐ (LowDP = scY cov 2.8–7) covered samples. Second, variant calling was performed among six moderately covered samples (ModDP = scY cov 17–18.5) and third among all 56 samples (reindeer, caribou, and moose). Sample details including grouping information are given in Table S1.

Obtained variants were filtered according to the following criteria (Figure S2): First, complex and multiallelic variants were removed from the variant list. Next, remaining variants were normalized with bcftools (v. 1.10.2‐105‐g7cd83b7; Danecek et al., 2021). The variants located on the mcY or nonY regions (Table S2) and the first/last 50 bp of each contig were removed. In the final step small insertion deletions (indels) were removed and only single nucleotide polymorphisms (SNPs) were kept for further analysis.

To generate a list of good‐quality moose variants, the filtered joint variant file consisting of 21,645 SNPs defined among all reindeer samples and the moose, was stringently filtered further (Figure S2). SNPs showing both the alternative and the reference allele (based on the number of reads reported for alleles in the vcf file) in more than two samples, and those with missing genotypes were excluded. Finally, remaining SNPs were filtered based on sample depth group, by keeping only variants covered with the following ranges: 1.5–2.5 for vLowDPmean samples; 3–7 for LowDPmean samples; 10–30 for ModDPmean samples. After applying these criteria, a collection of 192 moose SNPs was retained.

#### 2.5.3 Genotyping

A merged variant file consisting of high‐quality SNPs ascertained in the reindeer/caribou datasets and the moose private SNPs was created (Figure S2). Those 4265 SNPs were genotyped in the 56 samples using freebayes in haploid mode with “‐‐use mapping quality ‐‐min‐mapping‐quality 25 ‐‐min‐base‐quality 20” parameters. After filtering complex and multiallelic variants and normalizing the remaining variants, 3928 SNPs were obtained in 55 *R. tarandus* and a moose. Next, for each SNP the number of samples carrying both alleles, the alternative and the reference, was defined and the SNP was discarded if the number was >5. Finally, mean coverage depth values were estimated across the above‐mentioned three depth categories for each SNP. Only the SNPs which were in the following depth criteria according to depth group were kept: 2–8 for vLowDPmean; 3–10 for LowDPmean; 8–30 for ModDPmean.

#### 2.5.4 Creating the Y chromosomal phylogeny

Allelic states at 813 good quality Y‐chromosomal SNPs remaining after filtering were utilized to construct the initial draft parsimony tree in MEGAX (10.2.4; Kumar et al., 2018). After grouping the samples in the vcf file according to the phylogenetic structure, all variants were examined to determine their placement in the phylogeny, as described in Bozlak et al. (2023). From the variant list, 163 variants were removed due to one of the following categories: assembly errors, normalization failure by bcftools, confusion caused by missing calls, multi‐nucleotide polymorphisms, variants in repetitive sequences, variants determined in only one sample in heterozygous state, and recurrent variants. The final set of 650 variants (545 defined in 55 *R. tarandus* and 105 in the moose, Table S4) were used to construct the ultimate parsimony tree by utilizing MEGAX. In addition, a maximum likelihood (ML) tree was constructed with RAxML (v. 8.2.11) based on GTRGAMMA mode with 1000 bootstrap replicates. For determining the positions of the 650 final variants on OX460344.1, the 30 bp flanking regions were extracted and mapped to OX460344.1 by bwa aln “‐n 0.02 ‐l 200” parameters.

#### 2.5.5 Creating a haplotype network

To create a Y haplotype network with Eurasian samples, only the variants defined in 38 Eurasian samples and a single caribou sample (used as an outgroup) were selected from the variant list, and the missing positions were imputed based on the phylogeny as described in Bozlak et al. (2023). A median‐joining network with 346 variants was created in PopArt (v. 1.7; Leigh & Bryant, 2015). The samples and variants used in the network are indicated in Tables S1 and S4, respectively.

#### 2.5.6 Estimating the mutation rate

As a calibration point, we focused on the haplogroup (HG) “Y‐EB‐A2.B1” (Table S4) as the youngest EuroBeringian HG detected in North American *Rangifer* samples Al‐D‐ATC‐1 and Ca‐W‐WC‐14. We fixed the basal node of this HG (the calibration point “CP” in Figure 2b) to 11 kya, as this was the last possible natural contact of the EuroBeringian lineages of North America and Eurasia (Elias et al., 1996). We estimated the mutation rate by determining the average number of mutations occurring after this node in each branch. The length of the scanned region was determined to be the scY regions on “reindeerY1320” not including the first and last 50 bp of the contigs, resulting in a total length of 1,234,870 bp. A generation time of 6 years was assumed for reindeer based on Dussex et al. (2023). The mean number of 14.01 mutations after 11,000 years (1833 generations) in the scanned region revealed a mutation rate of 0.6187 × 10^−8^ per site per generation, or 0.1031 × 10^−8^ per site per year.

#### 2.5.7 Estimating coalescence times

We dated the branching points in the Y tree with BEAST2 (v. 2.7.3; Bouckaert et al., 2019). First an xml file was created in BEAUTI, by giving the 545 Y chromosomal SNPs detected only in *R. tarandus* lineages and the proportion of invariant sites to the program. HKY – gamma site heterogeneity was employed as the substitution model, with the mutation rate estimated as 0.1031 × 10^−8^ mutations per site per year. Lower and upper mutation rates were estimated as 0.0773 × 10^−8^ and 0.1546 × 10^−8^ considering generation times 8 and 4 years, respectively. Coalescent Constant Population as tree prior and strict clock model as molecular clock type were utilized. A total of 20,000,000 MCMC runs were generated. Finally, the tree was estimated using BEAST2 and subsequently combined with TreeAnnotator to derive the split dates and upper/lower ranges.

#### 2.5.8 Diversity analysis

Arlequin (v. 3.5.2; Excoffier & Lischer, 2010) was used to estimate the number of segregating sites among the sample groups defined in Table S1 from the fasta alignment file of 650 variants. Sequence diversity value ThetaW in scY region (1,360,096 bp) was estimated among groups according to Nei (1987) using R (v. 3.6.2).

### 2.6 mtDNA analysis

The trimmed reads from the 56 WGS samples used for Y analysis were mapped to the 16,358 bp mitochondrial reference sequence (MZ353654.1). Mapping and filtering processes were performed as described for Y variant ascertainment. The resulting bam files were converted to fasta files using angsd (v. 0.934; Korneliussen et al., 2014) with the following parameters: ‐doFasta 2 ‐doCounts 1 ‐setMinDepth 3 ‐minQ 20 ‐minMapQ 20. The resulting fasta files were merged and aligned using clustalo (v. 1.2.4; Sievers et al., 2011). After removing the 926 bp D‐loop region, a parsimony tree was constructed using MEGAX. In addition, a ML tree was estimated by RAxML based on GTRGAMMAI mode with 1000 bootstrap replicates.

## 3 RESULTS

### 3.1 Reindeer MSY contigs de novo assembly

**FIGURE 1:**
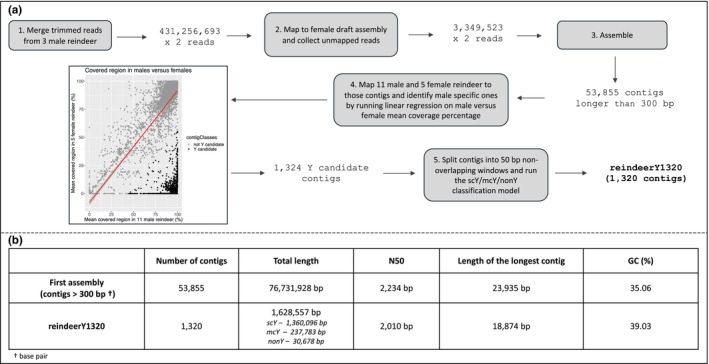
MSY assembly – strategy and results. (a) Flowchart showing the generation of the assembly and results. The plot shows the average percentage of each contig (in total 53,855) covered in males versus females. (b) Basic statistics of the first assembly and the final contig set “reindeerY1320.” For reindeerY1320 the length of single‐copy‐Y (scY), multi‐copy‐Y (mcY), and non‐Y is given.

We generated a reindeer draft Y‐assembly from short read sequencing data by mapping paired‐end Illumina reads from the genomes of three male reindeer to a published female reference genome (Li et al., 2017). A de novo assembly of all unmapped reads (6,699,046 paired reads) resulted in 64,821 contigs with a total length of 76.8 Mb. As shown in previous studies (Deng et al., 2020; Felkel, Vogl, et al., 2019; Wallner et al., 2017) such de novo contigs are a mosaic of MSY sequences and autosomal or X chromosome insertions not represented in the reference genomes. To extract the Y‐linked sequences, we mapped WGS reads from male and female reindeer to the de novo assembled contigs and defined 1324 Y candidates with a contig length longer >300 bp (see workflow and assembly results in Figure 1). We divided those contigs into 50 bp windows and based on mapping coverage depth in males and females, each window was classified as either single copy Y (scY), multicopy Y (mcY), or not typically Y (nonY), with a probabilistic model (Felkel, Vogl, et al., 2019). Four contigs that consisted mostly of nonY windows were discarded. On the remaining “reindeerY1320” draft Y‐assembly (GCA_963856025, Table S2), 27,789 50‐bp‐windows, in total 1,360,096 bp in length, were estimated as scY. Examples for mapping pattern are given in Figure S1.

Aligning the reindeerY1320 contigs to a recently released reindeer Y assembly (OX460344.1) generated from a Svalbard reindeer with a total length of 63 Mb (Dussex et al., 2023), revealed that 705 reindeerY1320 contigs (total length of 800 kb) could be placed (Table S3, Figure S1). Among those, 524 contigs (a total length of 551 kb/523 kb scY) mapped once while 181 contigs mapped multiple times on OX460344.1. The majority of the single match contigs (484) contained at least 90% scY region. Notably, the reindeerY1320 contigs were all located in a region spanning 53 and 63 MB on OX406344.1, and contigs with a single match were all placed in a circumscribed region between 57 and 59 Mb. The sex determining SRY gene is annotated in that region, as are three out of the other six single copy genes annotated on reindeer Y assembly OX406344. This result shows that our assembly strategy mainly captures sequences from an old stratum of the X‐degenerate region of the *Rangifer* Y chromosome.

Furthermore, 615 contigs (total length 739 kb/701 kb scY) could not be placed on OX460344.1 (Table S3). The unplaced contigs showed unequivocal male specific mapping results in all our samples, including the Svalbard reindeer in our sample set (Figure S1), indicating that they do represent reliable Y chromosomal sequence content which is not represented on OX460344.1.

A similar observation, namely that tailored enrichment of Y reads prior to the assembly step revealed Y sequence content not presented in a continuous Y assembly, was also made in horses (Felkel, Vogl, et al., 2019; Felkel, Wallner, et al., 2019). Conversely, mapping three male and three female reindeer to the OX460344.1 revealed that while 14 of the 63 Mb were covered in males, only 2–4 Mb had a coverage depth expected in scY regions (Table S1), while the majority of the regions covered had a much higher read depth, characteristic for multicopy regions. Overall, the comparison proved the validity and power of our strategy to assemble a significant proportion of Y reference sequence for unambiguous variant ascertainment.

### 3.2 Y and mtDNA topologies of *Rangifer tarandus*

**FIGURE 2:**
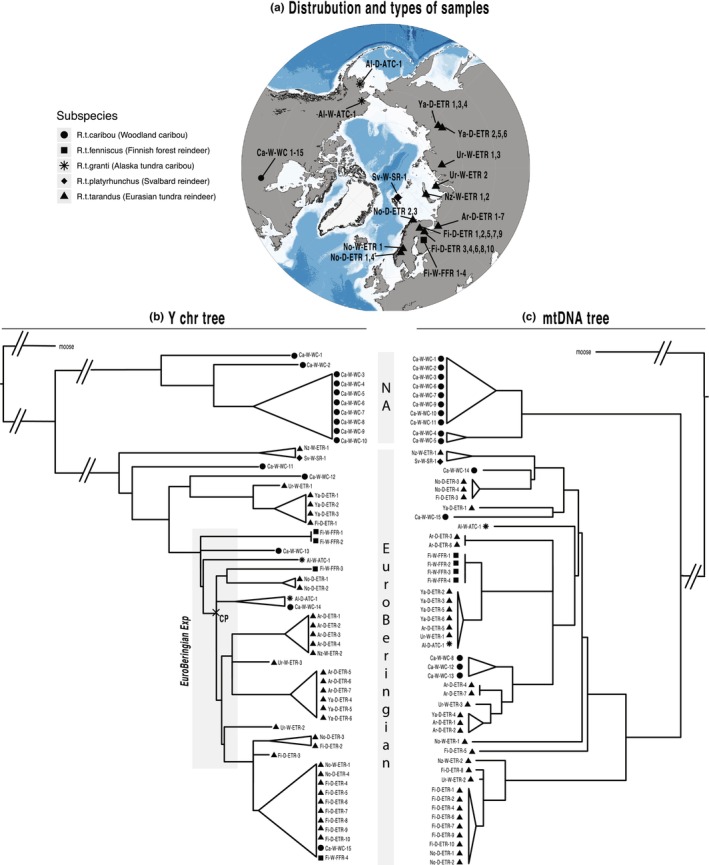
Distribution and types of sampled individuals, Y‐chromosomal and mtDNA trees. (a) Geographic regions of sample collection were plotted with ggOceanMaps package in R (4.2.2.). Different shapes refer to subspecies. Sample names reflect sampling location – type of animal (wild/domestic) – subspecies. Sample details are given in Table S1. (b, c) Y‐chromosomal and mtDNA parsimony trees of 55 male *Rangifer tarandus* samples rooted with a moose. The two well‐separated clades (NA and EuroBeringian) are marked. Trees are collapsed for major haplogroups (uncollapsed versions are in Figure S3). The proposed EuroBeringian Expansion event in Y‐chromosomal tree, related to population growth after the LGM is shown in the gray area and the calibration point (CP) for dating is marked. Maximum likelihood (ML) trees with bootstrap values are given in Figure S4.

We analyzed WGS data from 55 *Rangifer tarandus* males that represented five subspecies and a wide range of territories, from Norway to Sakha region (Yakutia), some Arctic Islands as well as the American continent (Figure 2a, Table S1).

Within *Rangifer*, we ascertained 545 high‐quality SNPs and those resulted in 50 Y‐chromosomal HTs (Table S4). In the whole mtDNA sequences (excluding the D‐loop) of the same individuals, we detected 39 HTs based on 458 SNPs (Table S5). We created Y and mtDNA parsimony trees and in both we discriminated two distinct clades (Figure 2b,c). One clade was formed by 10 woodland caribou from Canada (called as clade North American “NA”), while the second (“EuroBeringian”) comprised all Eurasian reindeer, the Svalbard reindeer, two Alaska tundra caribou as well as several woodland caribou. Notably, in both trees, Y and mtDNA, 10 out of 15 woodland caribou showed NA‐HTs. Only nine out of these ten individuals clustered into NA at both loci, while Ca‐W‐WC‐8 and Ca‐W‐WC‐11 carried NA at only one locus. Our mtDNA topology was in perfect concordance with the recently published comprehensive mtDNA topology including ancient and historic samples (Hold et al., 2024), underlining the representativeness of our sample set.

A sudden emergence of lineages was evident in EuroBeringian as well as NA clades in both phylogenies. Such “star‐like topologies” arise, when from a putative ancestral haplotype many new mutations have a high probability of surviving. They indicate that a lineage divergence event occurred at certain time points – the reason for that could have been an expansion of the population or an adaptive radiation. In the reindeer Y phylogeny, most notable was a prominent polytomy in the EuroBeringian Y clade, which we designated as an “EuroBeringian Expansion” sign (marked in Figure 2b).

The 45 males, that made up the EuroBeringian Y and mtDNA clades, represented all five subspecies and the collection included domesticated and wild animals from all over Eurasia, North America, and Arctic Islands (Figure 2). The deepest branch in the EuroBeringian Y clade was formed by the Svalbard reindeer and a wild Novaya Zemlya tundra reindeer (Sv‐W‐SR‐1, Nz‐W‐ETR‐1), highlighting the uniqueness of the two Arctic Island reindeer. In contrast, the deepest EuroBeringian mtDNA branch was formed by 10 domestic reindeer from Fennoscandia and two wild individuals, one from the Russian Ural Region and the other from Novaya Zemlya. Remarkably, EuroBeringian Y and mtDNA HTs were also detected in Alaska tundra caribou and some woodland caribou, but these mostly formed deep‐splitting private branches (Figure 2b,c).

### 3.3 Dating the emergence of Y‐chromosomal haplogroups

**FIGURE 3:**
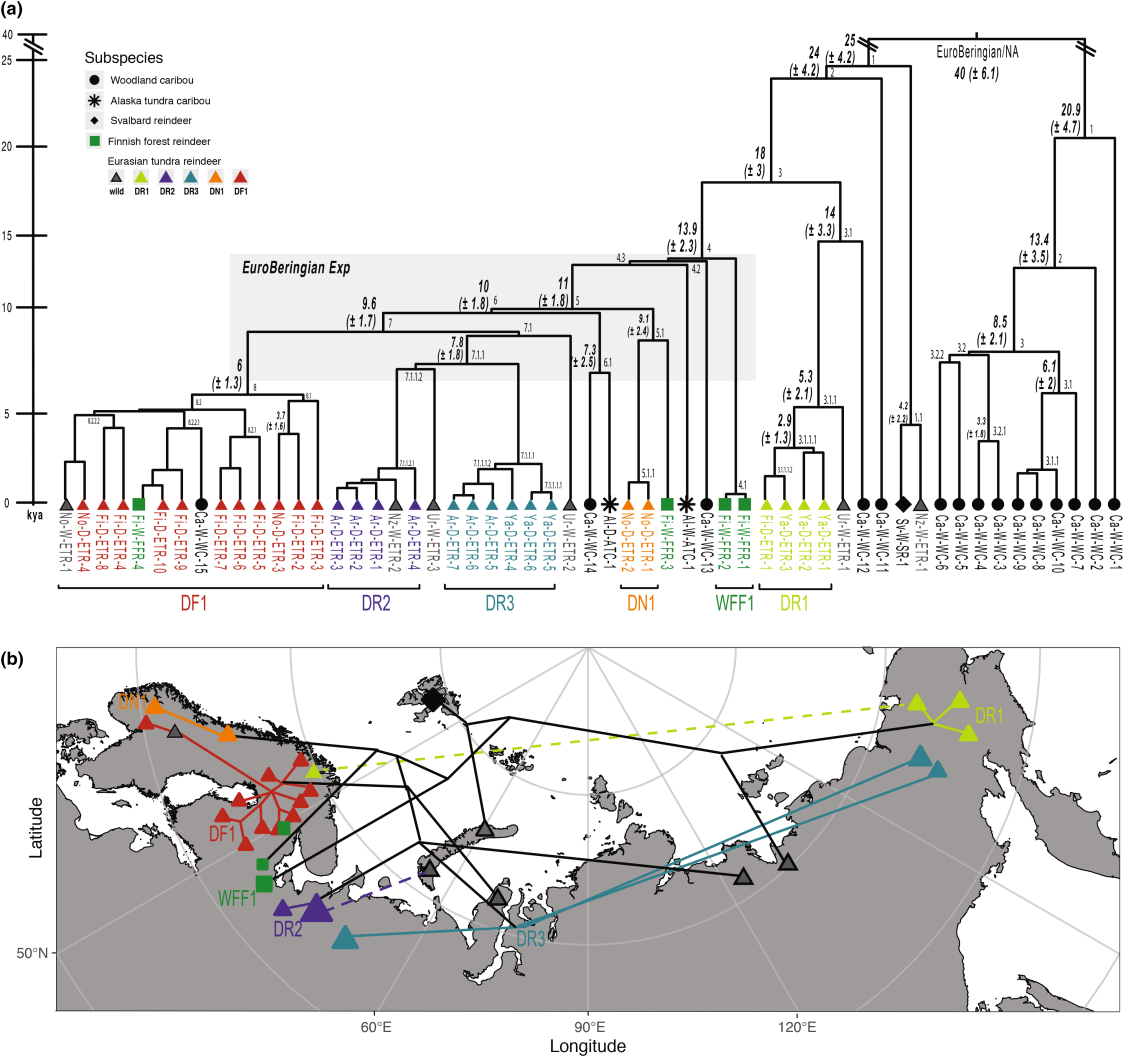
Y‐chromosomal tree with split dates estimated by BEAST and illustration summarizing the distribution of Y‐HTs in Eurasian samples. (a) Dating estimates are given on each node with 95% confidence intervals in parenthesis. Branching points after the EuroBeringian/NA are named hierarchically and the full information is provided in Table S6. The five haplogroups (HG) including mostly domesticated animals (DR1, DR2, DR3, DN1, DF1) and the Finnish forest reindeer HG (WWF) are marked. (b) Icons represent the HTs, with size proportional to the number of carriers. The combination of icon colors and shapes refers to subspecies and type (wild/semi‐domestic). Lines delineate the HT topology (full topology given in Figure S5), whereas dashed lines link HGs observed in geographically distant locations.

We took advantage of the unbiased variant ascertainment from WGS data and estimated divergence times of Y HTs under the assumption of a molecular clock. As a calibration point, we concentrated on EuroBeringian haplogroup (HG) “Y‐EB‐A2.B1” (Table S4) as the youngest HG shared between Eurasian reindeer and caribou from North America (Al‐D‐ATC‐1 and Ca‐W‐WC‐14). The last possible natural contact of the EuroBeringian lineages in North America and Eurasia was around 11 kya, as the Bering Bridge was no longer passable by land after that time (Elias et al., 1996). We estimated a mutation rate per site per year by setting the basal branching point of this HG (“CP”, Figure 2b) to 11 kya and averaged the number of mutations that occurred after this timepoint. Based on the mutation rate of 0.1031 × 10^−8^ /site/year in our setup, the separation of the two major Y clades (NA and EuroBeringian) was estimated to be 40 ± 6.1 kya (Figure 3a, Table S6). The deepest split in the EuroBeringian clade leading to the Svalbard/Novaya Zemlya was dated to 25 ± 4.2 kya and the deepest split in the NA clade was in a similar timeframe (20.9 ± 4.7 kya). The EuroBeringian Expansion was dated between 13.9 ± 2.3 and 9.6 ± 1.7 kya, whereas the expansion in NA (HG NA‐B2) was slightly later dated to 8.5 ± 2.1 kya. Within the EuroBeringian clade the reindeer from Fennoscandia formed a demarcated HG with signs of a sudden expansion of lineages (DF1 in Figure 3a). The most recent common ancestor (MRCA) of DF1 was estimated to be 6 ± 1.3 kya.

### 3.4 First Y insights into the population history of wild and domesticated Eurasian reindeer

In Eurasian reindeer, we defined five circumscribed Y HGs (Figures 3 and S5), whereby two were detected mostly in domesticated animals from Fennoscandia (DF1, DN1) and three in Russia (DR 1–3; Figure 3a,b). Strikingly, all five HGs had wild samples from Ural regions as sister branches and the estimated MRCA for the five HGs ranged from 1 ± 1 kya (DN1) to 6 ± 1.3 kya (DF1). HG DF1 was the most diverse among the five HGs based on the ThetaW value (1.10E‐05; Table 1). It was not only predominant in domesticated tundra reindeer from Finland but also observed in a domesticated and a wild animal from Norway, besides a caribou and a Finnish forest reindeer. On the other hand, HG DN1 was detected in only two domesticated reindeer from Norway. The Russian HGs DR1 and DR2 were restricted to Eastern and Western Russian domesticated reindeer respectively (except one sample from Finland clustering in DR1), while HG DR3 was detected in domesticated animals across geographically distant Russian regions (Figure 3 and Table 1). HG DR1 had an MRCA of 2.9 ± 1.3 kya and comprised three domestic samples from Yakutia and one from Finland and it had the highest ThetaW diversity value among Russian HGs (Table 1). In North‐Western Russia (specifically Arkhangelsk) we detected HG DR2, the least diverse Russian HG, and HG DR3, which was also found in Yakutia.

**TABLE 1:** Y‐diversity values in Eurasian samples.

Group	Number of samples	ThetaW
Eurasia domesticated	27	2.00E‐05
Eurasia wild (wo Svalbard)	10	3.50E‐05
Domestic haplogroups		
Y‐DF1	14	1.10E‐05
Y‐DR1	4	6.00E‐06
Y‐DR2	5	1.40E‐06
Y‐DR3	6	2.80E‐06
Y‐DN1	2	1.40E‐06

*Note*: Groups are indicated in Table S1.

Interestingly, the individuals clustering into the five well separated Y‐HGs showed, with some exceptions, similar grouping on the mtDNA tree. For instance, most of the domesticated reindeer from Fennoscandia clustering into DF1, formed a discrete HG also on the mtDNA tree. Likewise, also in congruence to the Y, domesticated reindeer from Russia clustered in several different mtDNA HGs which were all clearly distinct from the Fennoscandian mtDNA HG.

Three Finnish forest reindeer formed two private, early separated Y branches (MRCA to their closest neighbor 13.9 ± 2.3 kya and 9.1 ± 2.4 kya) in the EuroBeringian clade (HG WFF1 and sample Fi‐W‐FFR‐3), while the fourth forest reindeer (Fi‐W‐FFR‐4) clustered with domestic tundra reindeer from Finland into DF1 (Figures 3 and S5). In contrast, the six wild tundra reindeer in our dataset were dispersed across the EuroBeringian Y clade. Three wild tundra reindeer from Ural region (Ur‐W‐ETR1‐3), formed long private branches with MRCA to their closest neighbors between 5.3 ± 2.1 (youngest) and 9.8 ± 1.9 kya (oldest). One individual from Novaya Zemlya (Nz‐W‐ETR‐1) clustered with the Svalbard reindeer and they formed the deepest branch of EuroBeringian Y clade, as already mentioned above. Their MRCA was estimated far back in time, around 4.2 ± 2.2 kya. Contrary, the second wild Novaya Zemlya male (Nz‐W‐ETR‐2) and the single wild reindeer from Norway (No‐W‐ETR‐1) clustered with domesticated tundra reindeer from Western Russia (DR2) and Fennoscandia (DF1), respectively (Figures 2b and 3a).

## 4 DISCUSSION

Here, we present the first Y‐chromosomal phylogeny of *R. tarandus*, based on a de novo generated draft Y‐assembly and publicly available WGS data. The strategy for generating the assembly was based on short‐read WGS data of males not mapping to a female reference genome. In total, we revealed 1.6 MB Y chromosome sequence and out of these, 1.3 Mb were scY regions which were suitable to use as a reference for accurate variant ascertainment from short‐read sequencing data. The scY regions assembled for reindeer were comparable in size to ones' constructed in other species with a similar strategy; for example 0.5 Mb in sheep (Deng et al., 2020), 2.3 Mb in Bactrian camel (Felkel, Wallner, et al., 2019) and 1.4 Mb in horse (Wallner et al., 2017).

The total 1.3 Mb Y region facilitated the ascertainment of 545 SNPs in *Rangifer*. The variation obtained was sufficient in length to resolve the HT phylogeny, although we see differences in the topologies obtained via parsimony, maximum likelihood and Bayesian inference in groupings supported only by a small number of SNPs (Figures 2, 3 and S4).

Furthermore, we created the mtDNA tree without the D‐loop for our sample set. Until very recently (Hold et al., 2024), mtDNA studies in *Rangifer* were limited to either relying only on the control region (Heino et al., 2021; Røed et al., 2008) or particular genic regions (Yannic et al., 2014). Our objective here was to create robust Y and mtDNA phylogenetic trees across five subspecies and determine stable, parsimony informative markers for HT screening.

The hierarchic single locus Y tree, based on 55 samples from five subspecies, revealed that woodland caribou and EuroBeringian reindeer are clearly differentiated as evidenced by their deep splits in both phylogenies. The Y topology is therefore in agreement with established knowledge of two lineages based on mtDNA (Flagstad & Røed, 2003; Hold et al., 2024; Taylor et al., 2021) and autosomal (Dussex et al., 2023; Wu et al., 2024; Yannic et al., 2014, 2018) data.

Hence, the Y confirmed the hypothesis of their isolation by the Laurentide ice sheet (Hewitt, 1996; Yannic et al., 2014). However, the split time of the two Y clades was estimated around 40 ± 6.1 kya which is substantially younger than the 110 kya split time calculated from autosomal (Wu et al., 2024) and the 70kya calculated for the split of mtDNA clades (Hold et al., 2024). It is important to keep in mind that we used the last possible contact between EuroBeringian lineages over the continents (Figure 2b) to calibrate the Y phylogeny. Hence, our estimates are the latest possible timeframes for emergence of the Y‐HTs.

Notably, in both EuroBeringian and NA Y clades, we detected a sudden emergence of lineages around 14–7 kya and 10–6 kya respectively. These findings are in line with the different timeframes of the deglaciation in Eurasia and North America (Clark et al., 2009, 2012), and they are in concordance with suggested *Rangifer* populations expansions after the LGM on both continents (Flagstad & Røed, 2003; Weldenegodguad et al., 2020; Yannic et al., 2014).

In the EuroBeringian Y clade, the individual from Svalbard defined the deepest branch, together with a wild tundra reindeer from Novaya Zemlya. This is consistent with the unique clustering of the Svalbard reindeer on autosomal and mtDNA in previous studies (Dussex et al., 2023; Gravlund et al., 1998; Kvie et al., 2016; Pokharel et al., 2023). But beyond that, the deep joint branching of the Svalbard and Novaya Zemlya Y lineages substantiate the hypothesis of an early colonization of the Svalbard archipelago from the EuroBeringian population (Hold et al., 2024). Furthermore, the MRCA of Svalbard and Novaya Zemlya Y HTs was dated to 4.2 ± 2.2 kya and this estimate is only slightly younger than the split date of two lineages based on autosomal data (which was 6.2 kya in Dussex et al., 2023) and mtDNA (8 kya in Hold et al., 2024).

One striking signal in both phylogenies was the circumscribed grouping of Fennoscandian tundra reindeer (Figure 2b,c). The demarcation of Fennoscandian reindeer was shown previously based on mtDNA (Flagstad & Røed, 2003; Hold et al., 2024; Røed et al., 2008) and autosomal data (Pokharel et al., 2023; Weldenegodguad et al., 2020; Wu et al., 2024). An expanding population from an isolated refugium south of the European ice sheet after the post‐glacial recolonization was assumed as the source of present Fennoscandian reindeer (Flagstad & Røed, 2003; Røed et al., 2008). Our Fennoscandian Y and mtDNA topologies also provide clear evidence for a punctual expansion of lineages in the past. But while on mtDNA the Fennoscandian HG formed the deepest branch in the EuroBeringian clade, we did not detect an analogous early branching HG on the Y. Instead, the Fennoscandian Y‐HG “DF1” branched off from an inner node of the EuroBeringian clade. The expansion of DF1 was dated to 6 ± 1.3 (Figure 3a) and can therefore be linked to post LGM movements. Furthermore, the sister groups to DF1 (DR2, DR3, Ur‐W‐ETR‐2, and Ur‐W‐ETR‐3) were found in wild and domestic Russian tundra reindeer. Hence, Y data showed no evidence of an “old lineage” deriving from Central Europe as proposed by Flagstad & Røed (2003), but rather indicated an East Asian origin of most extant Fennoscandian patrilines.

Furthermore, the resolved Y topology provided initial insights into the paternal origin of some particular *Rangifer* populations such as Finnish forest reindeer and domesticated herds. Finnish forest reindeer, one of the recognized *Rangifer* subspecies, exists nowadays only in Finland and North‐Western Russia. Due to several natural and non‐natural causes such as predators, overhunting or the forestry industry, Finnish forest reindeer populations have been declining in the last years (Panchenko et al., 2021). We detected two private Y‐lineages in Finnish forest reindeer (WFF1 and Fi‐W‐FFR‐3), while one individual clustered with Fennoscandian domestic reindeer (Figure S5). Previous mtDNA studies revealed already two distinct mtDNA HGs in Finnish forest reindeer: one unique to the Finnish forest reindeer whereas the second one shared with domesticated tundra reindeer from Fennoscandia and Russia. The latter HG has also been detected in ancient samples from the same regions (Heino et al., 2021). Interestingly, four Finnish forest reindeer in our dataset carried the identical mtDNA HT, and this HT shared its MRCA with an HG mostly formed by samples from Arkhangelsk, Ural, and Yakutia (Figures 2, S3 and S4). Hence, the Y and mtDNA data herein substantiate a unique origin of the Finnish forest reindeer as indicated also in the study by Pokharel et al. (2023).

The single Finnish forest reindeer clustering with domestic tundra reindeer in the Y (HG DF1) might carry the signal of genetic interaction between the populations. However, previous autosomal data analysis of the same four males did not reveal any influence from domesticated tundra reindeer (Pokharel et al., 2023; Weldenegodguad et al., 2023). Altogether we conclude that the DF1 HT in this Finnish forest reindeer is the signal of an introgressed Fennoscandian tundra male way back in time (dated around 0.8 ± 0.7 kya), and this is not detectable on the autosomes anymore. Overall, our Y data underline the uniqueness of the Finnish forest reindeer, with at least two private Y lineages in the population.

In our dataset, we had 27 domesticated EuroBeringian reindeer and those formed five Y HGs (Figure 3). These HGs were distributed over the EuroBeringian clade suggesting that several wild lineages underwent domestication, which is in line with the proposed two or more independent domestication events in a recent study based on autosomal data (Wu et al., 2024). In Fennoscandian domesticates, two Y HGs, DF1 and DN1, were identified. DF1, mostly composed of animals from Finland and Norway, emerged around 8–6 kya and is likely the signal of reindeer that migrated to Fennoscandia from East Asia after the EuroBeringian Expansion. Along similar lines, recent studies argue (Bjørklund, 2019; Salmi & Seitsonen, 2022) that the reindeer involved in the transition to large‐scale herding in Fennoscandia (from limited domestic reindeer in a hunting livelihood before) actually were “imported” from the East, from Russia, and not domesticated from wild Fennoscandian herds. Correspondingly, in Sámi oral history the herding of domestic reindeer with ancestry from the East was considered more prestigious than those of local semi‐domesticated ones from Fennoscandia, as the animals from the East were more docile than those tamed from local Fennoscandian herds (Salmi & Seitsonen, 2022).

In addition, we found a unique domestic male lineage in Norway (DN1). Albeit there is evidence of reindeer in West Norway around 30 kya based on archeological data (Valen et al., 1996), DN1 HTs cannot descend from this ancient population because DN1 emerged much later. Given the topology, DN1 definitely origins from populations that moved to Fennoscandia during or after the EuroBeringian Expansion.

In domesticated reindeer from Russia, we identified three Y HGs (DR1, DR2, and DR3) with their estimated expansion dates (Figure 3) in the range of the earliest recorded domestication sign observed in Siberia around 2 kya (Losey et al., 2021). DR1 was primarily detected in East Russian, particularly Yakutia, DR2 mainly in North‐Western Russia, specifically the Arkhangelsk region, and DR3, was found in Arkhangelsk and Yakutian reindeer. Y data confirmed the genetic differentiation between Fennoscandian (DF1, DN1), Eastern Russian (DR1) and Western Russian (DR2) domesticated populations shown from mtDNA (Røed et al., 2008) and autosomal data (Pokharel et al., 2023; Weldenegodguad et al., 2020; Wu et al., 2024) before. Here, we postulate HG DR1 as the signal of an Eastern Russian domestication, from which most of the reindeer herding cultures in the Eurasian Arctic evolved (Losey et al., 2021; Stammler, 2005). The clustering of Yakutian DR1 carriers together with caribou, Alaskan, and Ural‐wild samples in the mtDNA tree further indicates that DR1 originated in the Beringian/Yakutian region.

HG DR2, which was shared between Arkhangelsk and one wild male from Novaya Zemlya, might derive from another domestication event in Western Russia. However, it is important to note that three out of the four males from Arkhangelsk in DR2 carried the same HT (Figure S3), indicating the dominance of a distinct male lineage in our samples from this region. Besides, DR2 Arkhangelsk males clustered with DR3 Arkhangelsk and with Yakutian reindeer on the mtDNA tree. This may suggest that although DR2 was domesticated in Western Russia, it also has an Eastern Eurasian origin. This is in line with anthropological, linguistic and archeological theories of the Eastern origin of reindeer herding, which also assume the cradle of domestication as well as the roots of many Siberian indigenous peoples in the Sayan‐Altai mountains and the Trans‐Baikal territory (Mark, 1970; Pomishin, 1990; Vainshtein, 1980). For HG DR3, which was found in Eastern and Western Russian samples in equal numbers, the topology also suggested an Eastern origin (Figures 3 and S5).

In Arctic reindeer herding, wild males often unintendedly reproduce with domestic females (Klokov, 1997), a practice which herders such as the Nenets or Eveny try to prevent as it may lead to disintegration and loss of the domestic herd. Besides, in some cultures using wild males to reproduce with domesticated females is a common practice (Anderson et al., 2017; Oehler, 2020). The Y could provide evidence for such cases when domesticated reindeer carry wild Y‐HTs or form single long branches, in our limited dataset. Overall, we did not observe such a pattern in our data, with domestic samples more likely to be grouped together in clusters. However, due to our small sample size, this finding should be interpreted with caution.

Another intriguing insight into breeding or trading practices was evident from recently emerged Y‐HGs discovered in geographically distant regions (Figure 3). Detection of the Yakutian HG DR1 in a Fennoscandian sample (Fi‐D‐ETR‐1), HG DR2 in Novaya Zemlya, and the Fennoscandian HG DF1 in a caribou (Ca‐W‐WC‐15) clearly highlighted the long‐distance movement of domesticated reindeer. Reindeer exchange within and beyond kinship network is common among all reindeer herding cultures in the Arctic (Stammler, 2005; Ziker, 1998), and has also crossed territories and boundaries over large distances (Anderson, 2000; Vitebsky, 2005).

For the first time, we are able to compare Rangifer Y and mtDNA topologies and both showed remarkably similar patterns; like the separation of NA and EuroBeringian clades, the circumscribed Fennoscandian HGs and the clustering of domesticated animals. A congruence of the two uniparental markers is rarely observed in domestic species, where different reproduction dynamics of the sexes cause differences between Y and mtDNA signals (Achilli et al., 2012; Deng et al., 2020; Felkel, Vogl, et al., 2019). The agreement in reindeer points to rather similar mating and dispersal patterns in the sexes. Also, domesticated reindeer exhibits much greater Y sequence diversity, than other domesticated mammals. Although ThetaW is still higher in the wild than in domesticated reindeer in our dataset, we revealed multiple domestic haplogroups dispersed throughout the entire EuroBeringian clade. This is also contradictory to what is observed in domestic species such as horses, sheep, and goat, where only a few haplotypes have been selected, developed, and distributed through intense breeding (Bozlak et al., 2023; Deng et al., 2020; Xiao et al., 2021). In addition, most reindeer in our panel carried a unique Y HT defined by private variants, even when clustering in the domestic HGs (Table S4; Figure S5). The pronounced Y‐chromosomal sequence diversity in domesticated reindeer can be either interpreted as reindeer domestication is still in its early stages (Baskin, 2000), or it may reflect the unique breeding strategies applied by herders based on the unique biology and needs of the animals and humans in a specific natural and cultural environment (Beach & Stammler, 2006; Stammler & Takakura, 2010). The broad spectrum of Y HTs in domestic reindeer are in concordance with Wu et al. (2024), who detected comparable levels of genetic diversity and linkage disequilibrium in wild and domestic reindeer populations.

The robust MSY and whole mtDNA topologies of *Rangifer tarandus* confirmed several previous findings and allowed new insights into the populations' history and present. However, it is worth to note that our outcomes depend on several technicalities such as the type and depth of sequencing data, the size of the region scanned, the approaches used for variant ascertainment and filtering (Jobling & Tyler‐Smith, 2017). Here, we merged short read WGS data from different studies, which had several multitudes difference in sequencing depth, we focused on a limited 1.3 Mb MSY region, used a single variant caller and applied strict filtering of variants to determine the true positive variants. Altogether, these may cause an underestimation of the branch lengths in our tree. In the future, adjusting the current limitations will lead to more informative variants and a better resolution of the phylogeny. Apart from technical limitations, we still underestimate the *Rangifer* HT spectrum because of the ascertainment sample set. We tried to represent the current subspecies and ecotypes in our first Y phylogeny, and we had samples from one of the largest wild reindeer populations of Eurasia, Taymyr (Savchenko et al., 2020), but we still lack numerous wild and domestic populations. In order to reveal a more complete picture and answer many open questions, a more representative, comprehensive sample collection is essential, as well as more thorough integration of biological samples with indigenous oral histories about the origin of reindeer. In the future, implementing ancient data could reveal a precise view on emergence and spread of lineages through time and space (Bozlak et al., 2023; Librado et al., 2021; Wutke et al., 2018), which is key to explain the dispersal of Y and mtDNA lineages and the domestication‐related questions.

Overall, we provide valuable polymorphic Y and mtDNA markers for *Rangifer* species that can be straightforwardly used to determine HT composition of populations. Such data will contribute to a better understanding of the dispersal of lineages, the diversity in populations and the admixture between groups. The resolved Y and mtDNA trees may provide direction for protecting the diversity and integrity of *Rangifer tarandus*, by allowing a more fine‐grained analysis of the differences among subspecies and/or ecotypes, the domesticated and wild populations, as well as the domestic types, that have particular importance for the diversity of human–animal livelihoods in specific habitats in a warming Arctic.

## AUTHOR CONTRIBUTIONS

**Elif Bozlak:** Conceptualization (equal); data curation (equal); formal analysis (lead); investigation (lead); software (lead); visualization (lead); writing – original draft (equal); writing – review and editing (equal). **Kisun Pokharel:** Data curation (equal); writing – review and editing (supporting). **Melak Weldenegodguad:** Data curation (equal); writing – review and editing (supporting). **Antti Paasivaara:** Data curation (equal); writing – review and editing (supporting). **Florian Stammler:** Investigation (supporting); writing – review and editing (equal). **Knut H. Røed:** Investigation (supporting); writing – review and editing (equal). **Juha Kantanen:** Conceptualization (equal); data curation (equal); project administration (equal); writing – review and editing (equal). **Barbara Wallner:** Conceptualization (equal); funding acquisition (lead); investigation (supporting); methodology (supporting); project administration (lead); writing – original draft (equal); writing – review and editing (equal).

## CONFLICT OF INTEREST STATEMENT

The authors declare no competing interest.

## BENEFIT‐SHARING STATEMENT

Benefits from this research accrue from the sharing of our data and results on public databases as described above.

## Supporting information


Figure S1.
Figure S2.
Figure S3.
Figure S4.
Figure S5.

Table S1.

Table S2.

Table S3.

Table S4.

Table S5.

Table S6.

## Data Availability

Whole genome sequencing data are publicly available. Bioproject and Biosample Information is given in Table S1. The Y‐chromosomal contig assembly “reindeerY1320” is available on ENA (GCA_963856025). The Y‐chromosomal and mtDNA mappings, the multiple fasta alignment file of mtDNA sequence and the mapping of three male and three female reindeer to OX460344.1 are available in Zenodo (10.5281/zenodo.10394887; 10.5281/zenodo.10394706; 10.5281/zenodo.11092583). Computational codes used in the study are available at https://github.com/bozelf/Y‐mtDNA_reindeerPhylogenyPipeline.
