# Supplementary material for: Inferences about the population history of Rangifer tarandus from Y chromosome and mtDNA phylogenies
Source: Ecol Evol. 2024 Jun 10;14(6):e11573. doi: 10.1002/ece3.11573 (PMC11164974; doi:10.1002/ece3.11573)
Supplement: Supplementary file 1 — Figure S1. Figure S2. Figure S3. Figure S4. Figure S5. [file ECE3-14-e11573-s005.pdf]

Supplemental Information for

Inferences about the population history of Rangifer tarandus from the first Y-chromosome and mtDNA phylogenies

Elif Bozlak, Kisun Pokharel, Melak Weldenegodguad, Antti Paasivaara, Florian Stammler,
Knut H. Røed, Juha Kantanen, Barbara Wallner

Supplementary Figures

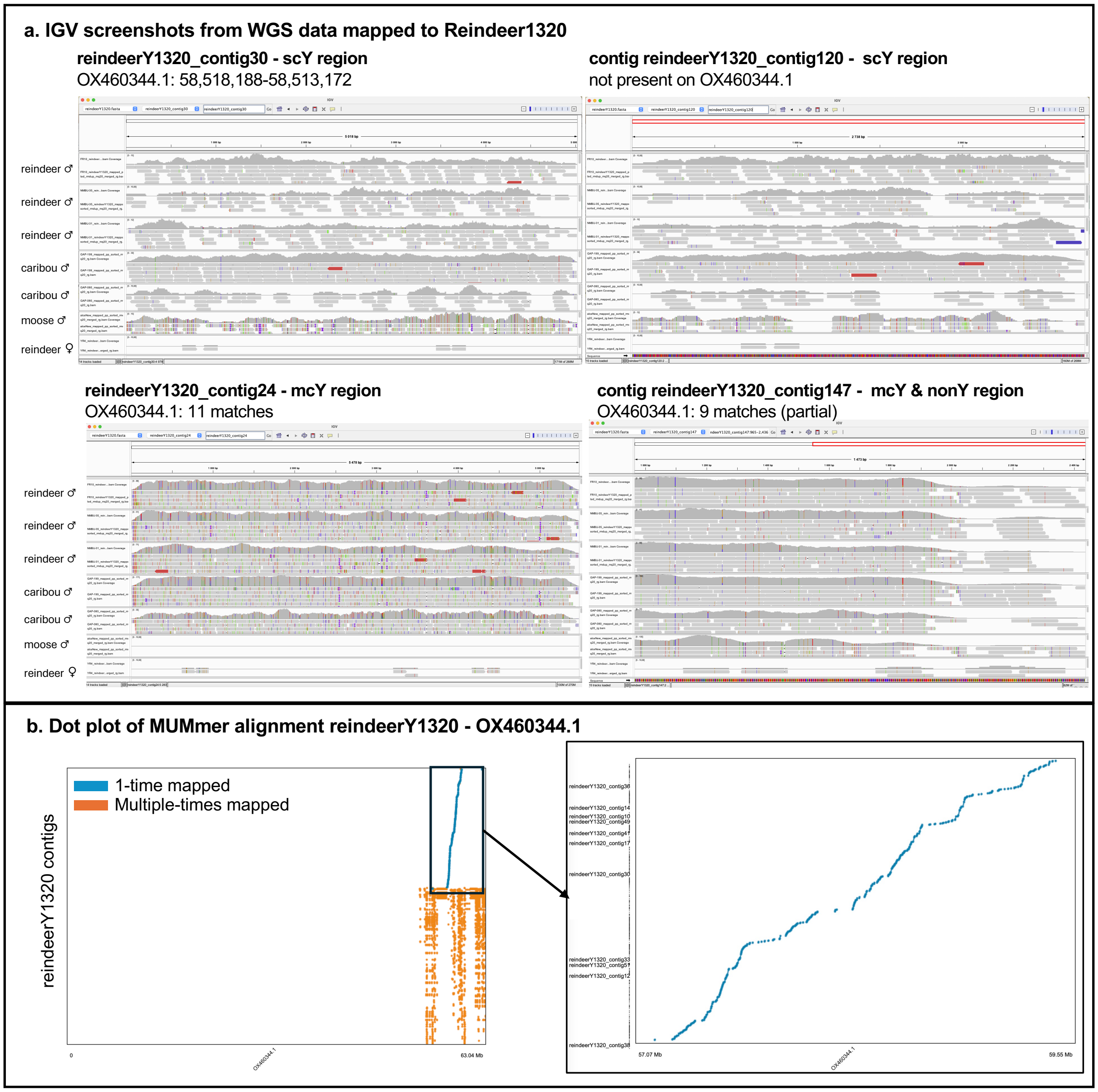

Figure S1: a. IGV screenshots of 3 male reindeer, 2 male caribou, 1 male moose and a female reindeer mapped to ReindeerY1320. The images show characteristic MSY mapping pattern in scY/mcY and nonY regions. While contig 30 was located on OX460344.1, no homology was detected for contig 120. Coordinates for ReindeerY1320 on OX460344.1 are given in Table S3.
b. Dot plot alignment results of reindeerY1320 versus OX460344.1 based on MUMmer. Full length OX460344.1 is on the X- and reindeerY1320 contigs are on the Y-axis. The left panel shows the full-length alignment whereas the right panel shows only the reindeerY1320 contigs with a single match on OX460344.1.

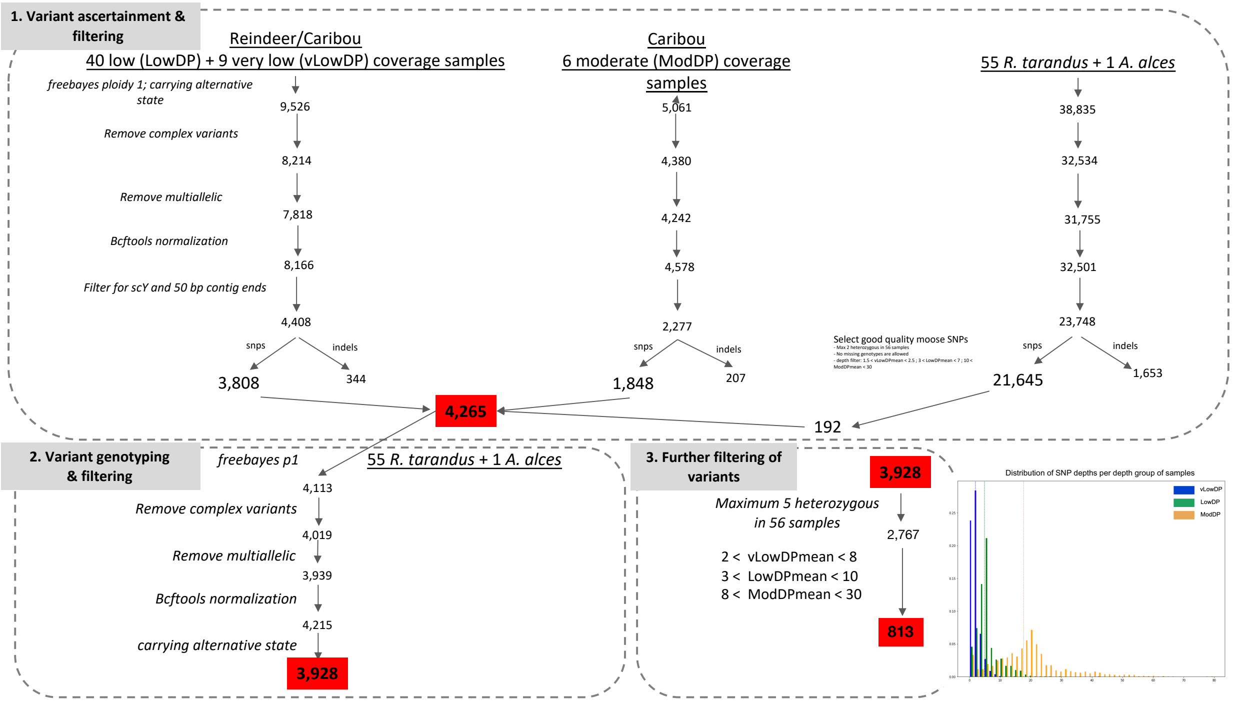

Figure S2: Flowchart of Y-chromosomal variant analysis workflow and results after ascertainment, filtering, and genotyping. The samples' sequencing depth status (vLowDP, LowDP, ModDP) are given in the Table S1. In the bottom right graph, the distribution of coverage depth over 2,767 Y-chromosomal SNPs is plotted for the sequencing depth groups.

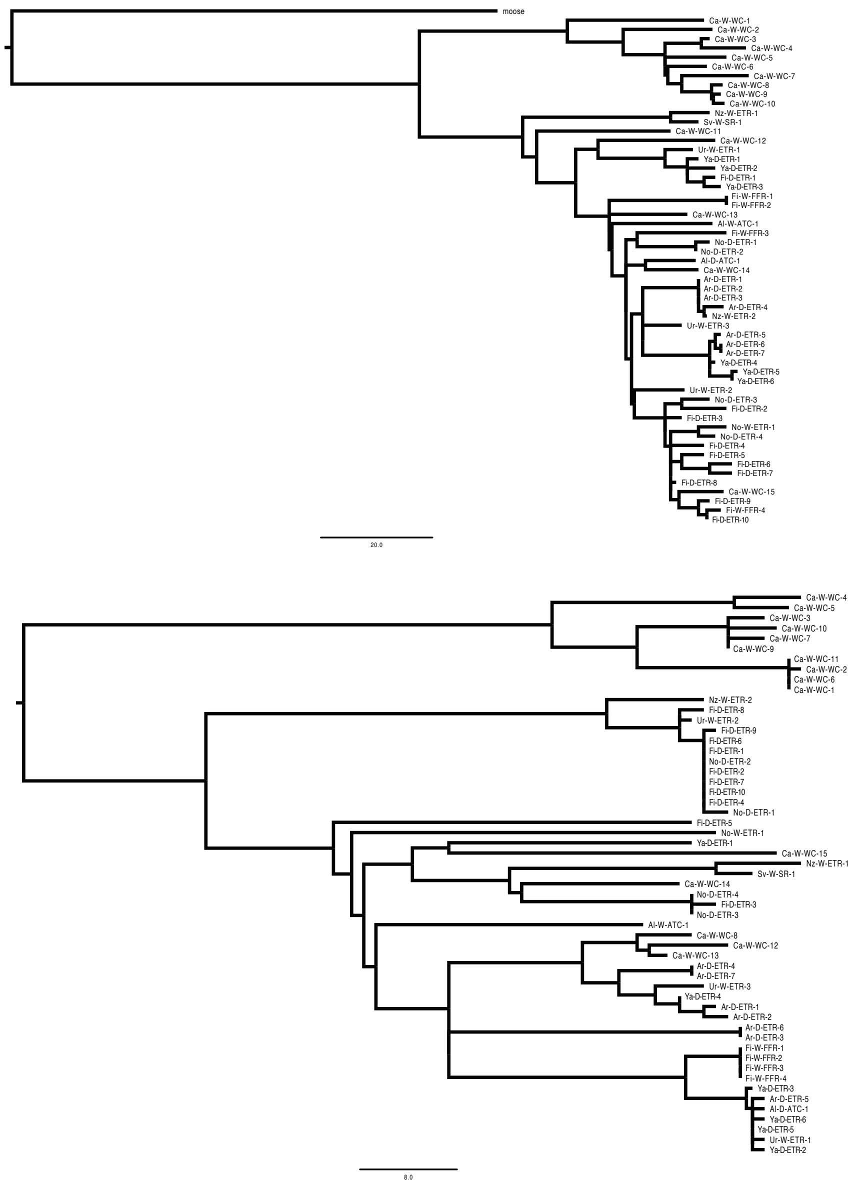

Figure S3: Related to Figure 2. Not collapsed version of the Y (upper) and mtDNA (lower) parsimony trees. The mtDNA tree is midpoint rooted to visualize the branches properly.

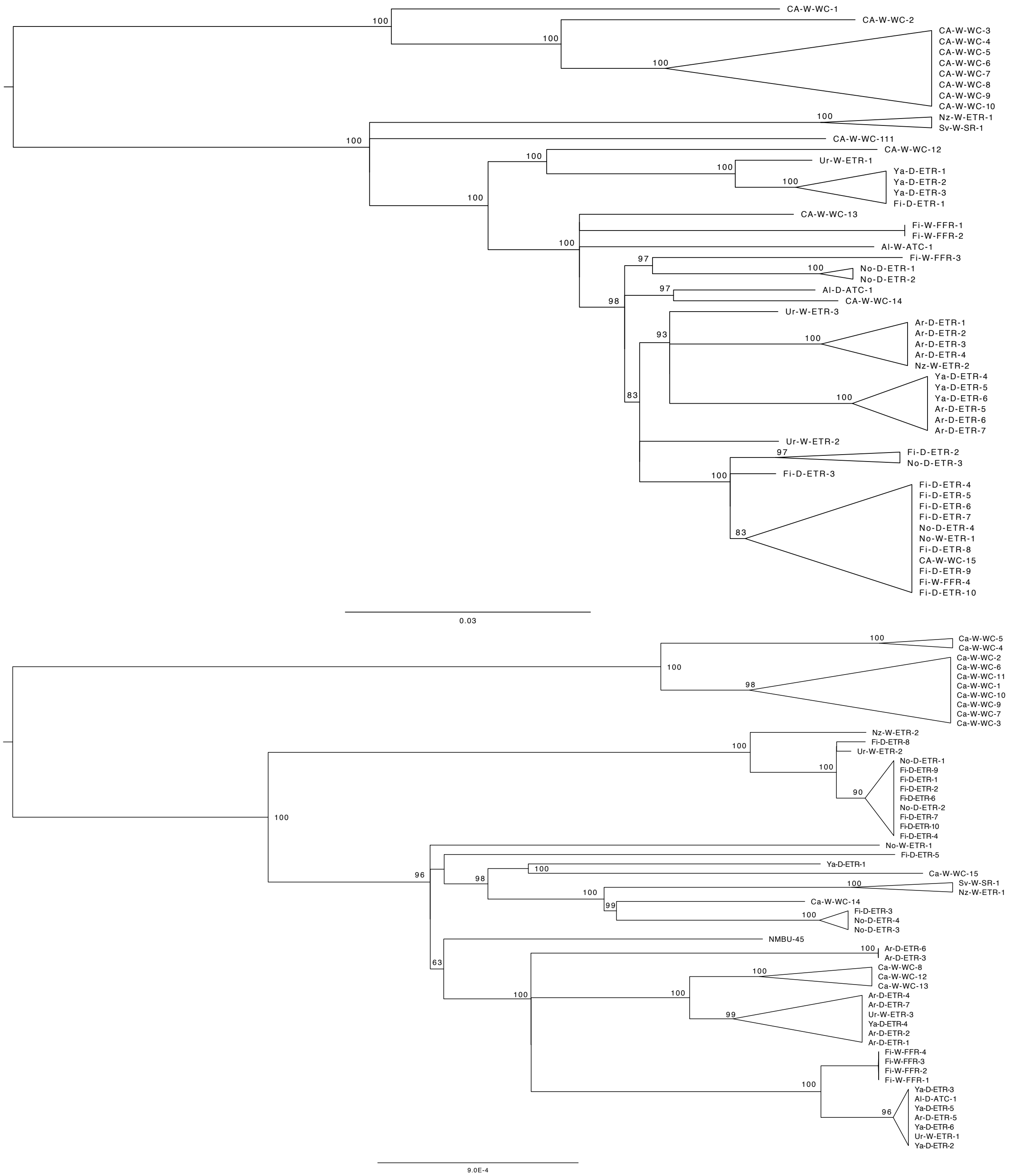

Figure S4: Y (upper) and mtDNA (lower) maximum likelihood trees of 55 *R. tarandus* samples with bootstrap values. Both trees are midpoint rooted.

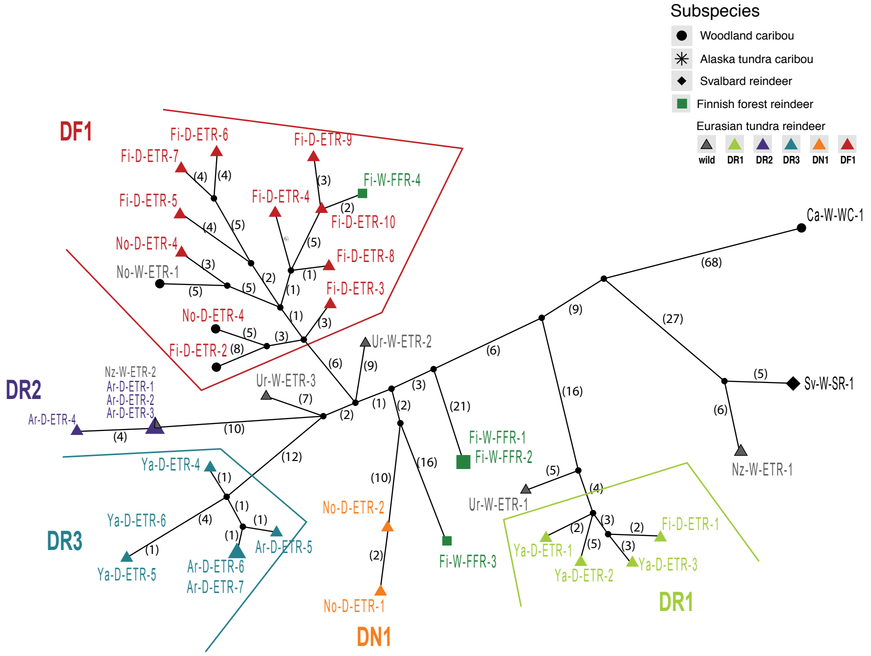

Figure S5: Y network of Eurasian reindeer samples based on 346 variants, rooted with a caribou (Ca-W-WC-1). The number of SNPs is given in parenthesis on the branches. Clades consisting of domesticated animals and the Finnish forest reindeer are marked. The combination of the colors and shapes represent the subspecies and the HG. Sample information is given in Table S1 and variant information in Table S3.

Supplementary Table Legends

Table S1: Samples metadata. The first sheet gives detailed information about all samples used in the study. Sample ids, details of sampling locations, raw and analyzed sequencing data statistics, and results of clustering analysis on Y and mtDNA are provided. The second sheet gives the results of WGS data from 9 caribou BLASTed against the *R. tarandus SRY* mRNA sequence.

Table S2: Assembly information. The first sheet gives the contig names and the lengths. The second sheet gives 50 bp scY windows defined on the contigs.

Table S3: BLAST results of reindeerY1320 versus OX460344.1. In the first sheet column A-E give summary statistics for each reindeerY1320 contig; column F gives how many variants were defined on each contig; columns G and H gives how many matches the contig had on OX460344.1 according to the BLAST alignment. The second sheet gives the BLAST output of reindeerY1320 and OX460344.1 with 95 percentage identity and 100 bp match filter.

Table S4: Y chromosomal variant table. The first sheet gives information on 650 variants used to create the Y chromosomal tree. Columns B-I give the information of each variant, including 30 bp flanking region. Columns J-BM shows the allelic states, depth and allelic depth of each sample including one moose and 55 *R. tarandus* samples for each variant. Derived allele status is shaded in grey. Columns BN-BP give the further information for each variant including their ancestral/derived states and network usage information, and the HT that they determine. In Columns BQ and BR for the 327 variants placed on OX460344.1, coordinates and their orientation on OX460344.1 is provided. Samples are ordered according to their haplogroups and variants are ordered hierarchically according to their position on the parsimony tree. Sheet 2 shows the 346 imputed variants used to create the network with 38 Eurasian samples and one caribou for Figure S5.

Table S5: mtDNA variant list providing information for mtDNA 458 variants. Columns A-B gives the variant information. Columns C-BE shows the base detected in each sample for the given variant. Column BF shows the HT the variant determines, if unambiguous. This column also shows if a variant is a 'recurrent mutation', which means the polymorphism is detected in different haplogroups. Samples are ordered based on their haplogroups and parsimony informative variants are sorted according to branch position on the tree. The recurrent mutations which couldn't be unambiguously assigned to the branches of the tree are given in the end of the list.

Table S6: Split dates estimated with BEAST. Estimated split dates and confidence intervals are provided. Node names match with Figure 3a.
